# miR-21 promotes NLRP3 inflammasome activation to mediate pyroptosis and endotoxic shock

**DOI:** 10.1038/s41419-019-1713-z

**Published:** 2019-06-12

**Authors:** Zhenyi Xue, Qing Xi, Hongkun Liu, Xiangdong Guo, Jieyou Zhang, Zimu Zhang, Yan Li, Guangze Yang, Dongmei Zhou, Huiyun Yang, Lijuan Zhang, Qi Zhang, Chao Gu, Juhong Yang, Yurong Da, Zhi Yao, Shuguang Duo, Rongxin Zhang

**Affiliations:** 1 Laboratory of Immunology and Inflammation, Department of Immunology, Key Laboratory of Immune Microenvironment and Diseases of Educational Ministry of China, Tianjin Key Laboratory of Cellular and Molecular Immunology, Tianjin Medical University, 300070 Tianjin, China; 2 Institute of Integrative Medicines for Acute Abdominal Diseases, Nankai Hospital, Tianjin, China; 3 Department of Genetics, School of Basic Medical Sciences, Tianjin Medical University, Tianjin, China; 4 Metabolic Disease Hospital & Tianjin Institute of Endocrinology, Tianjin Medical University, 300070 Tianjin, China; 5 Institute of Zoology, Chinese Academy of Sciences, 100101 Beijing, China; 6 Guangdong Province Key Laboratory for Biotechnology Drug Candidates, School of Life Sciences and Biopharmaceutics, Guangdong Pharmaceutical University, Guangzhou, China

**Keywords:** Cell death and immune response, Bacterial infection

## Abstract

miR-21 is aberrantly expressed, and plays a role in various types of tumors and many other diseases. However, the mechanism of miR-21 in LPS-induced septic shock is still unclear. In this study, we investigated the mechanism of miR-21 in LPS-induced pyroptosis and septic shock. Here, we show that miR-21 deficiency inhibited NLRP3, ASC, and caspase-1 expression, as well as inflammasome activation in myeloid cells from both mice and humans. We found that the NF-κB pathway was regulated by miR-21, and that A20 was a direct target of miR-21. Furthermore, miR-21 deficiency inhibited the ASC pyroptosome, which restrained caspase-1 activation and GSDMD cleavage, thereby preventing LPS-induced pyroptosis and septic shock. miR-21 deficiency resulted in an increase in A20, which led to decreased IL-1β production and caspase-1 activation. Caspase-1-mediated GSDMD cleavage was consequently decreased, which prevented pyroptosis in LPS-induced sepsis in mice. Our results demonstrate that miR-21 is a critical positive regulator of the NF-κB pathway and NLRP3 inflammasomes in pyroptosis and septic shock via A20. In addition, by analyzing published miRNA expression profiles in the Gene Expression Omnibus database, we found that the miR-21 levels in peripheral blood from patients with septic shock were elevated. Thus, miR-21 may serve as a potential treatment target in patients with septic shock.

## Introduction

The nucleotide-binding domain, leucine-rich repeat-containing receptor (NLR) family protein NLRP3 plays key roles in host defense, which can be activated by many pathogen-derived, environmental, and host-derived factors, including bacteria^1^, viruses^2^, fungi^3^, components of dying cells^1^, and crystal particles^4–7^. Secretion of proinflammatory IL-1β and IL-18 requires activation of inflammasomes to splice IL-1β and IL-18 precursors into mature forms^8^. Macrophages are the major source of pro-IL-1β and pro-IL-18, which are generally dependent on caspase-1 for maturation and secretion of the bioactive cytokine. Caspase-1 activation is controlled by different inflammasomes^9^. In addition, activated caspase-1 triggers a form of programmed necrosis known as pyroptosis.

Accumulating evidence has suggested that inflammasomes are involved in the pathogenesis of sepsis^10,11^. Inflammasomes trigger pyroptosis in a caspase-1-dependent manner^12,13^. In sepsis, pyroptosis promotes pore formation in the plasma membrane, leading to cell swelling and membrane rupture and resulting in leakage of abundant inflammatory factors out of the cell^10–15^. Caspase-1 and caspases-4/5/11 specifically recognize and then cleave gasdermin D (GSDMD), which is the core event in pyroptosis^16–18^. Caspase-1 also initiates pyroptosis by cleaving GSDMD. The role of GSDMD in pyroptosis is to trigger lethal sepsis and septic shock^18,19^.

Sepsis is defined as life-threatening organ dysfunction, and is caused by a several dysregulated responses to infection. Septic shock is a subset of sepsis with significantly increased mortality^20^. Many microRNAs (miRNAs) have been identified as key regulators of sepsis and septic shock. A previous study has reported that miR-21 is obviously elevated in the plasma of septic patients and the heart tissue of LPS-induced sepsis mice^21^. Although deregulations of miRNA expression are well described, the pathophysiological role of miRNAs in septic shock has not yet been fully defined.

Due to the key role of the NLRP3 inflammasome in LPS-induced septic shock, we investigated the function of miR-21 in regulating activation of the NLRP3 inflammasome. We found that miR-21 positively regulates activation of NLRP3 inflammasomes by negatively regulating A20 in macrophages, which triggers pyroptosis to promote LPS-induced septic shock through cleavage of GSDMD.

## Results

### miR-21 knockout inhibits NLRP3 inflammasome-mediated caspase-1 activation and IL-1β secretion in macrophages

**Fig. 1:**
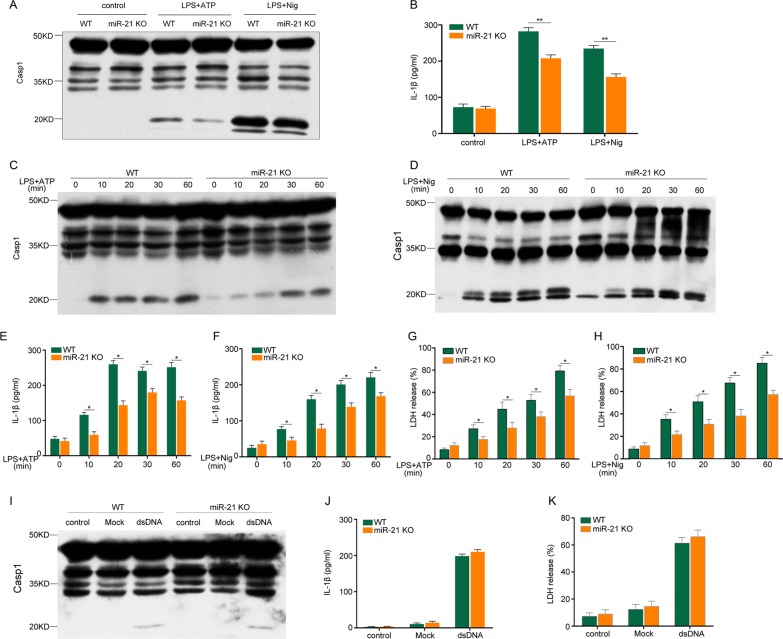
miR-21 deficiency inhibits NLRP3 but not AIM2 inflammasome activation and IL-1β secretion in macrophages. **a**, **b** BMDMs were primed with 500 ng/ml LPS for 4 h and then stimulated or not with 5 mM ATP or 20 mM nigericin for 30 min. Lysates were immunoblotted for caspase-1, and supernatants were analyzed for IL-1β. **c**–**h** BMDMs were primed with 500 ng/ml LPS for 4 h and stimulated with ATP or nigericin for different lengths of time. Lysates were immunoblotted for caspase-1 (**c**, **d**), and supernatants were analyzed for IL-1β (**e**, **f**) and LDH (**g**, **h**). **i**, **j**, **k** BMDMs were transfected with Lipo3000 (mock) or dsDNA as described in the Methods section. Lysates were immunoblotted for caspase-1 (**i**), and supernatants were analyzed for IL-1β (**j**) and LDH (**k**). The data represent the mean ± SD of one among three biological replicates, with three technical replicates each (*P < 0.05; **P < 0.01; Student’s *t* test)

To investigate the role of miR-21 in activation of the NLRP3 inflammasome, lipopolysaccharide (LPS)-primed BMDMs were challenged with ATP or nigericin. We found that miR-21 knockout (KO) inhibited caspase-1 activation and IL-1β secretion (Fig. 1a, b). Moreover, caspase-1 autoprocessing (Fig. 1c, d) and IL-1β secretion (Fig. 1e, f) caused by NLRP3 inflammasomes significantly decreased during ATP or nigericin treatment for 10 min and continued to decrease in a time-dependent manner. Induction of LDH-release-based cell death by caspase-1-dependent pyroptosis was weakened in miR-21^−/−^ macrophages (Fig. 1g, h). The increased reactivity of macrophages to inflammasomes was limited to NLRP3 inflammasomes because stimulation of AIM2 inflammasomes with dsDNA did not induce caspase-1 cleavage, IL-1β secretion, or pyroptosis in wild-type and miR-21^−/−^ macrophages (Fig. 1i–k). These data clearly indicate that miR-21 promotes IL-1β secretion and caspase-1 activation mediated by the NLRP3 inflammasome in mouse macrophages.

### miR-21 regulates NF-κB and NLRP3 inflammasome expression by targeting A20

**Fig. 2:**
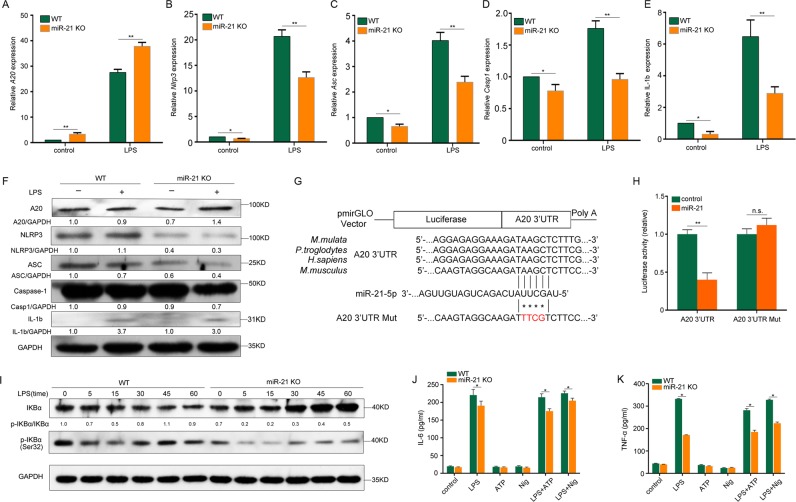
A20 is a functional miR-21 target for regulation of NF-κB and NLRP3 inflammasomes. **a**–**e** *A20* (**a**), *Nlrp3* (**b**), *Asc* (**c**), *Casp1* (**d**), and IL-1b (**e**) mRNA levels in LPS-treated WT and miR-21 KO BMDMs. **f** A20, IL-1b and NLRP3 inflammasome protein levels in LPS-treated WT and miR-21 KO BMDMs after 4 h. **g** Sequence alignment of the A20 3′-UTR with miR-21 across multiple species. **h** Activity of luciferase reporters containing WT or mutant A20 3′-UTRs that were used with miR-21 mimics or respective controls to cotransfect HEK-293T cells. **i** BMDMs were treated with 500 ng/ml LPS for different lengths of time. Cell extracts were immunoblotted for IKBα and p-IKBα. **j**, **k** WT and miR-21 KO BMDMs were left unstimulated (control) or stimulated with LPS (500 ng/ml), ATP (5 mM), or nigericin (20 mM) alone or stimulated with LPS (500 ng/ml) for 4 h and then treated with 5 mM ATP or 20 mM nigericin for 30 min. Culture supernatants were analyzed for IL-6 secretion (**j**) and TNF secretion (**k**). The data represent the mean ± SD of one among three biological replicates, with three technical replicates each (*P < 0.05; **P < 0.01; Student’s *t* test)

To determine the target of miR-21, we further detected the upstream regulatory factors of the NLRP3 inflammasome. In miR-21^−/−^ macrophages, *A20* mRNA expression was increased, and *Nlrp3*, *Asc, Casp1*, and *Il-1b* mRNA expression was decreased (Fig. 2a–e). Western blotting results also showed that the A20 protein level was upregulated, but that NLRP3, ASC, pro-caspase-1, and pro-IL-1b protein levels were downregulated in miR-21^−/−^ macrophages (Fig. 2f). To confirm that A20 is a direct target of mir-21, 293 cells were cotransfected with control or miR-21 mimic and a dual-luciferase reporter plasmid containing the WT A20 3′-UTR. The results showed that the activity of luciferase in miR-21 mimic-transfected cells was significantly decreased. However, miR-21 mimic had no significant effect on the activity of luciferase in cells transfected with the dual-luciferase reporter plasmid containing an A20 3′-UTR mutated at the mir-21-binding site (Fig. 2g, h).

NLRP3 inflammasome expression levels are regulated by the proinflammatory transcription factor NF-κB^22^. A20 negatively regulates LPS-induced NF-κB activation, which also influences the secretion of the NF-κB-dependent cytokines IL-6 and TNF in macrophages^23-26^. Inducible activation of NF-κB depends upon proteasomal degradation of NF-κB protein inhibitors (IkBs)^27^. In this study, we found that there was a dramatic decrease in p-IkBα levels following LPS stimulation for different time intervals in miR-21 KO cells, resulting in increased IkBα levels (Fig. 2i). In addition, miR-21 deficiency inhibited the secretion of the NF-κB-dependent cytokines IL-6 and TNF in macrophages (Fig. 2j, k).

**Fig. 3:**
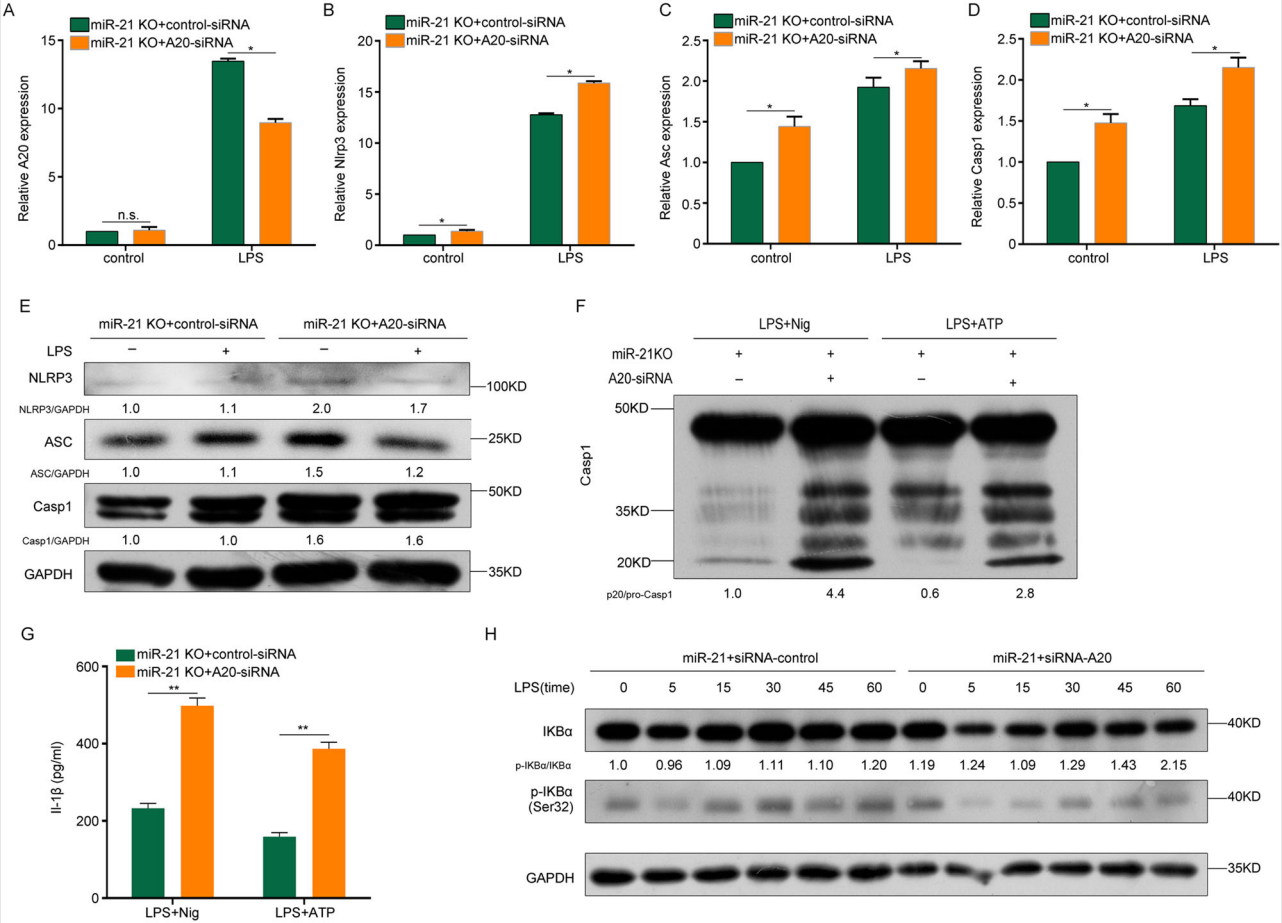
miR-21 regulates NF-κB and NLRP3 inflammasomes through A20. **a**–**d** *A20* (**a**), *Nlrp3* (**b**), *Asc* (**c**), and *Casp1* (**d**) mRNA levels in LPS-treated miR-21 KO BMDMs transfected with control or A20 siRNA. **e** Protein levels of NLRP3 inflammasomes in LPS-treated miR-21 KO BMDMs transfected with control or A20 siRNA. **f** Activation of caspase-1 in LPS-primed and ATP- or nigericin-treated miR-21 KO BMDMs transfected with control or A20 siRNA. **g** Supernatants were analyzed for IL-1β in (**f**). **h** BMDMs were treated with 500 ng/ml LPS for different lengths of time in miR-21 KO BMDMs transfected with control or A20 siRNA. Cell extracts were immunoblotted for IKBα and p-IKBα. The data represent the mean ± SD of one among three biological replicates, with three technical replicates each (*P < 0.05; **P < 0.01; Student’s *t* test)

We further confirmed the target of miR-21 using loss-of-function experiments. The mRNA results showed that A20 knockdown promoted *Nlrp3*, *Asc* and *Casp1* mRNA expression in miR-21 KO macrophages with or without LPS stimulation (Fig. 3a–d). Similarly, the NLRP3, ASC, and pro-caspase-1 protein levels were enhanced in A20 knockdown macrophages with or without LPS stimulation (Fig. 3e). In addition, caspase-1 processing and IL-1β secretion were increased in A20 knockdown macrophages with LPS priming and ATP or nigericin stimulation (Fig. 3f, g). Collectively, these data demonstrate that NF-κB and NLRP3 inflammasome expression was regulated by miR-21 through targeting of A20.

### miR-21 regulates NLRP3-mediated ASC pyroptosome formation by targeting A20

**Fig. 4:**
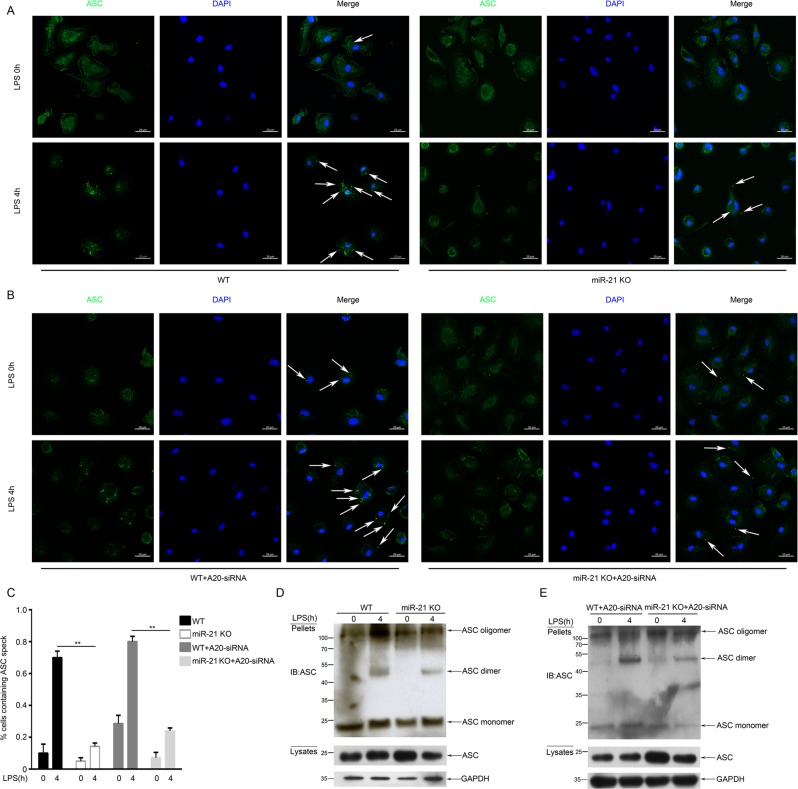
miR-21 regulates ASC pyroptosome formation. **a**, **b** Immunofluorescence microscopy of LPS-primed macrophages untreated or stimulated with ATP and then stained for ASC and DNA (with DAPI). Scale bars: 20 μm. **c** Percentage of macrophages containing ASC foci. The quantification represents the mean of three independent experiments, with at least 50 cells counted in each experiment. **d**, **e** ASC oligomerization and redistribution assay in peritoneal macrophages treated as in **a** and **b**. Immunoblot analysis of ASC in cross-linked pellets (upper panels) and in cell lysates (lower panels)

In addition to caspase-1 activation and IL-1β release, the ASC pyroptosome is another hallmark of inflammasome activation, which is considered to mediate caspase-1 activation^28^. In accordance with previous reports, the results of endogenous ASCs immunostaining showed that more than 70% of macrophages contained the ASC pyroptosome in the WT (70.4%) and A20 knockdown (80.1%) group after stimulation with LPS and ATP. Conversely, formation of the ASC pyroptosome was prevented in the miR-21 KO group (14.3% and 24.2%) (Fig. 4a–c). In addition, we isolated the ASC pyroptosome using a chemical method reported in previous studies^29^. We detected endogenous ASC pyroptosome in BMDMs via western blot analysis. The results showed that ASC proteins were cross-linked by DSS to form ASC dimers, trimers, and oligomers, and were mainly redistributed from the lysates into the pellets in the WT group compared with the miR-21^−/−^ group (Fig. 4d). Similarly, the capacity to form ASC pyroptosomes increased after A20 knockdown in the WT group, but decreased after A20 knockdown in the miR-21^−/−^ group (Fig. 4e). Thus, consistent with the confocal assay, ASC redistribution and oligomerization were suppressed in miR-21^−/−^ macrophages.

### miR-21 deficiency inhibits pyroptosis by preventing caspase-1-mediated cleavage of GSDMD

**Fig. 5:**
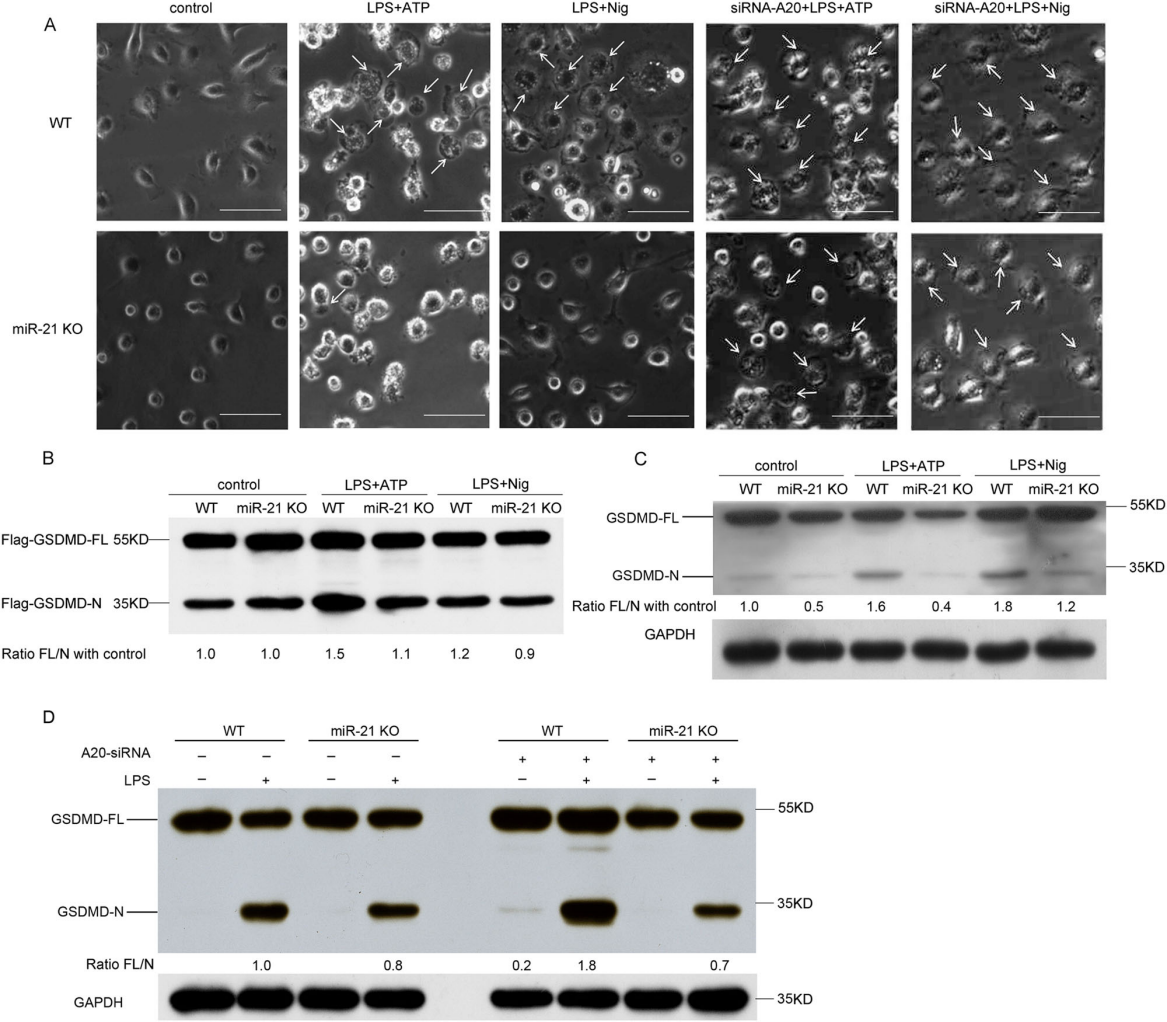
miR-21 deficiency inhibits pyroptosis by preventing cleavage of GSDMD. **a** Imaging assay of pyroptosis in WT and miR-21 KO BMDMs treated as indicated. Scale bars: 100 μm. **b** Immunoblotting demonstrates cleavage of GSDMD. WT and miR-21 KO BMDMs stably expressing Flag–GSDMD were stimulated as indicated. **c** Immunoblotting with mouse antibody for GSDMD demonstrates cleavage of GSDMD in WT and miR-21 KO BMDMs treated with ATP or nigericin. **d** Immunoblotting demonstrates cleavage of GSDMD in WT and miR-21 KO BMDMs treated with A20 siRNA. GSDMD-FL full-length GSDMD, GSDMD-N N-terminal cleavage products of GSDMD. All data are representative of three independent experiments

We further examined the role of miR-21 in canonical inflammasome-triggered pyroptosis. In miR-21^−/−^ cells, BMDM pyroptosis was blocked upon NLRP3 inflammasome activation triggered by LPS and ATP or nigericin (Fig. 5a). Dying cells presented a typical pyroptosis morphology, with cell swelling and membrane rupture. In contrast, pyroptosis was enhanced in A20-deficient WT and miR-21^−/−^ macrophages (Fig. 5a). Because GSDMD is only required for pyroptotic cell death and acts downstream of inflammatory caspases, GSDMD was cleaved in BMDM cells stimulated with LPS plus ATP or nigericin. A cleavage product corresponding to the N-terminal half of Flag–GSDMD was identified, and was decreased compared with the control (Fig. 5b). Similarly, the cleaved GSDMD N-terminus was decreased when miR-21 KO BMDMs were stimulated with LPS plus ATP or nigericin compared with control cells (Fig. 5c). We also found that knockdown of A20 promoted cleavage of GSDMD in WT cells, but this was not observed in miR-21 KO cells (Fig. 5d). These data demonstrate that miR-21 KO inhibits GSDMD cleavage-induced pyroptosis via targeting of A20.

### miR-21 deficiency relieves LPS-induced septic shock and organ damage

**Fig. 6:**
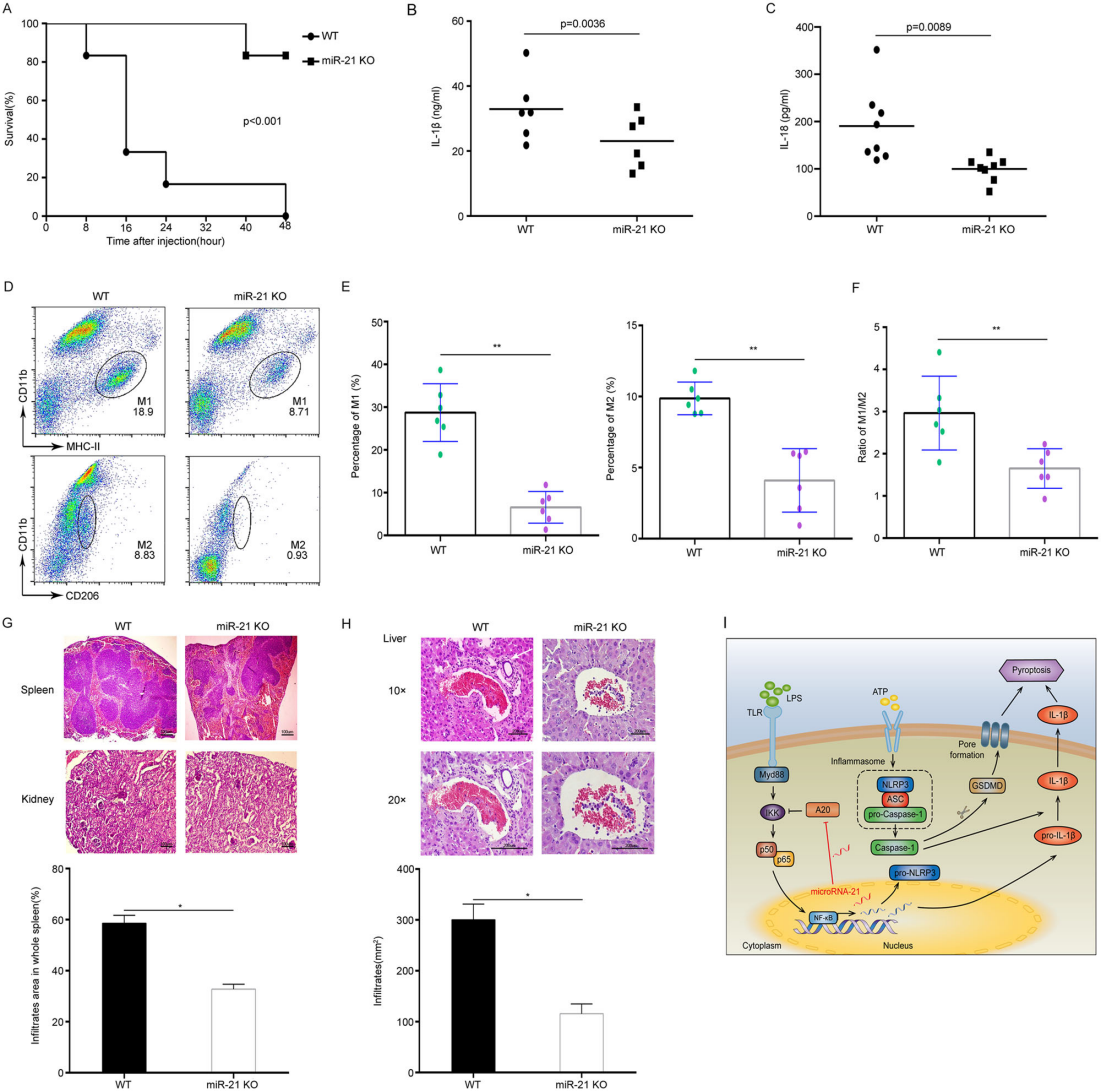
miR-21 deficiency relieves LPS-induced septic shock and organ damage. **a** Survival curve after LPS (35 mg/kg of body weight) was injected intraperitoneally into WT mice (*n* = 12) and miR-21 KO mice (*n* = 12). **b**, **c** Production of IL-1β and IL-18 in peritoneal lavage fluid 12 h after intraperitoneal injection of LPS (35 mg/kg of body weight) into WT mice (*n* = 6) and miR-21 KO mice (*n* = 6). **d** Flow-cytometry analysis of peritoneal M1 and M2 macrophages from WT and miR-21 KO mice treated with LPS. **e** The percentage of peritoneal M1 and M2 macrophages in WT mice (*n* = 6) and miR-21 KO mice (*n* = 6) treated with LPS. **f** The ratio of peritoneal M1 and M2 macrophages in WT mice (*n* = 6) and miR-21 KO mice (*n* = 6) treated with LPS. **g** Histological and quantitative analyses of spleens and kidneys from WT mice and miR-21 KO mice. Scale bars: 100 μm. **h** Histological and quantitative analyses of livers from WT mice and miR-21 KO mice. Scale bars: 200 μm. **i** The mechanistic model of miR-21-mediated inflammasome activation and pyroptosis

To confirm the function of miR-21 in activating NLRP3 inflammasomes in vivo, we studied the role of miR-21 in an LPS-induced septic shock mouse model. The results showed that miR-21 knockout led to much lower mortality rates than those observed in wild-type mice after peritoneal injection of LPS (Fig. 6a). Furthermore, IL-1β and IL-18 secretion levels were significantly decreased in the peritoneal lavage fluid from miR-21^−/−^ mice (Fig. 6b, c). The proportion of M1 and M2 macrophages was also obviously decreased in peritoneal lavage cells from miR-21^−/−^ mice (Fig. 6d, e), and the M1/M2 ratio was obviously decreased in miR-21^−/−^ mice (Fig. 6f). To determine the cause of death, we performed histological analyses of the internal organs. We observed fewer lesions in spleens, kidneys, and livers from miR-21^−/−^ mice, which also showed decreased inflammatory cell infiltration compared with WT mice (Fig. 6g, h). Collectively, these results demonstrate that miR-21 acts as a critical positive regulator of the NF-κB pathway to regulate NLRP3 inflammasome activation and pyroptosis through negative regulation of A20 (Fig. 6i).

### Role of miR-21 in human septic shock

**Fig. 7:**
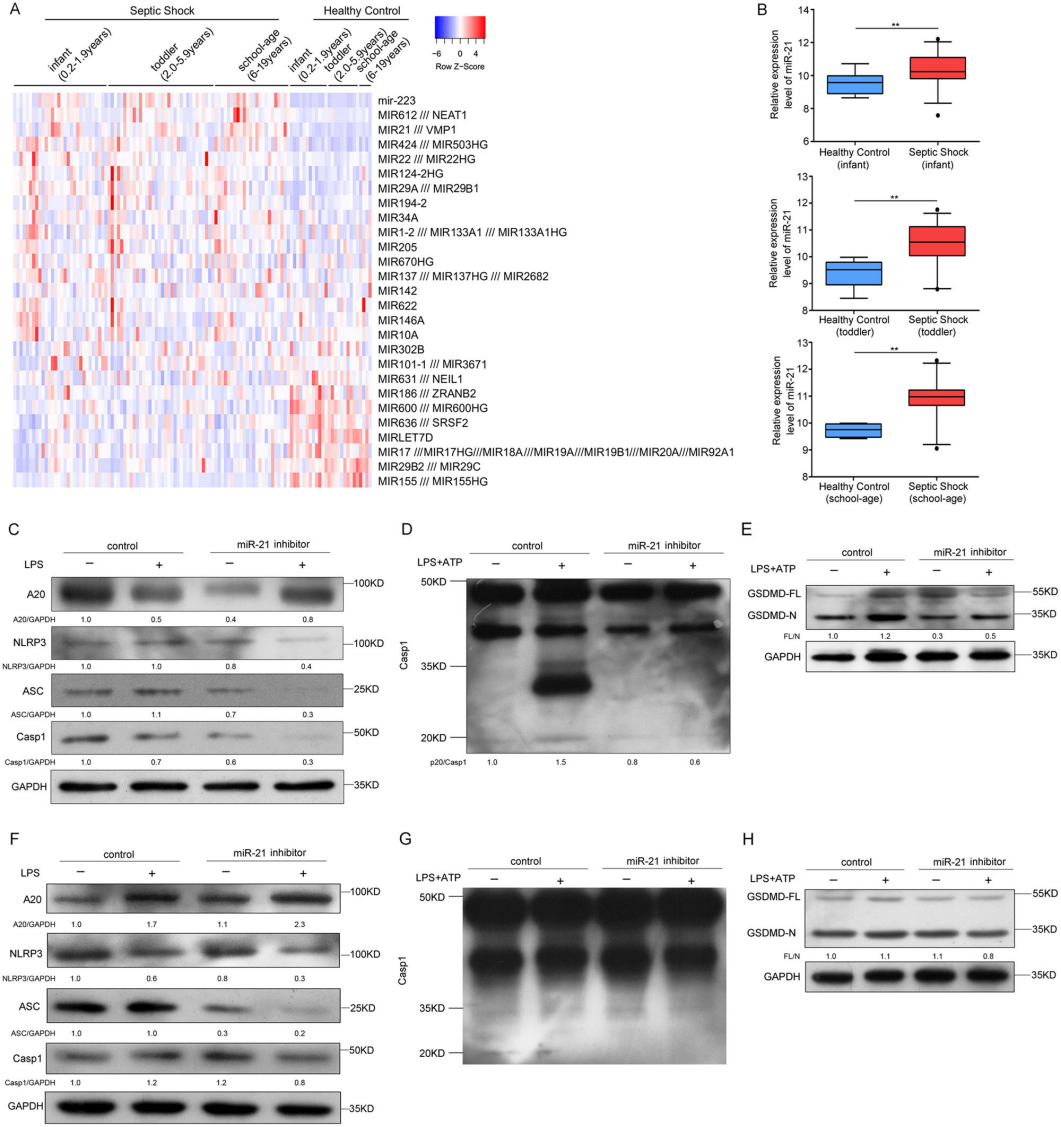
The role of miR-21 in human septic shock. **a**, **b** Analysis of one published human septic shock clinical data set (GSE26440) and quantification of miR-21 expression from corresponding datasets. **P < 0.01. The statistical significance of the differences was estimated using nonparametric two-sided Mann–Whitney tests. **c** PMA-differentiated THP-1 cells were transfected with control siRNA and a miR-21 inhibitor followed by stimulation with 500 ng/ml LPS for 4 h. Cell extracts were immunoblotted for A20 and NLRP3 inflammasomes. **d**, **e** PMA-differentiated THP-1 cells were transfected with control siRNA and a miR-21 inhibitor followed by stimulation with 500 ng/ml LPS for 4 h and treatment with ATP for 30 min. Cell extracts were immunoblotted for caspase-1 activation and GSDMD cleavage. **f** Human PBMCs were transfected with control siRNA and a miR-21 inhibitor followed by stimulation with 500 ng/ml LPS for 4 h. Cell extracts were immunoblotted for A20 and NLRP3 inflammasomes. **g**, **h** Human PBMCs were transfected with control siRNA and a miR-21 inhibitor followed by stimulation with 500 ng/ml LPS for 4 h and treatment with ATP for 30 min. Cell extracts were immunoblotted for caspase-1 activation and GSDMD cleavage

To evaluate the role of miR-21 in human septic shock, we searched original microRNA expression profile data in the Gene Expression Omnibus (www.ncbi.nlm.nih.gov/geo/) database for human septic shock. We downloaded GSE26440 expression data of samples from 88 children with septic shock and 26 normal controls from the Gene Expression Omnibus (GEO) database. Differentially expressed miRNAs in samples from patients with septic shock were analyzed in comparison with those in samples from normal controls. We found that miR-21 was obviously upregulated in septic shock patients in the clinical data set (Fig. 7a), including infants (0.2–1.9 year, *n* = 30), toddlers (2.0–5.9 years, *n* = 34), and school-age children (6–19 years, *n* = 24) (Fig. 7b). In miR-21 inhibitor-treated PMA-differentiated THP-1 cells, A20 protein levels were upregulated, but NLRP3, ASC, and pro-caspase-1 protein levels were downregulated (Fig. 7c). We next treated LPS-primed PMA-differentiated THP-1 cells with ATP and found that the miR-21 inhibitor blocked caspase-1 activation and cleavage of GSDMD (Fig. 7d, e).

Next, we stimulated human peripheral blood mononuclear cells (PBMCs) from healthy donors with LPS. Consistent with our findings in THP-1 cells, A20 protein levels were upregulated, but NLRP3, ASC, and pro-caspase-1 protein levels were downregulated (Fig. 7f). We further treated LPS-primed PBMCs with ATP and found that the miR-21 inhibitor prevented caspase-1 activation and cleavage of GSDMD (Fig. 7g, h). Therefore, miR-21 is also crucial for regulation of NLRP3 inflammasome activation and GSDMD cleavage in human cells. In general, these data demonstrate that the anti-inflammatory effect of miR-21 KO is conserved between mice and humans.

## Discussion

Previous studies have shown that certain miRNAs are abnormally expressed in sepsis patients, and some are associated with decreased survival, supporting their potential use as targeted biomarkers for early sepsis diagnosis and prognosis determination^30^. In addition to its role in various tumor types, miR-21 is aberrantly expressed and plays a role in LPS-induced septic shock^21^, but the mechanisms are still unclear. Our study showed that the proinflammatory effect of miR-21 may result from its ability to promote NLRP3 inflammasome activation, thereby promoting the secretion of IL-1β and cleavage of caspase-1 and GSDMD. Furthermore, miR-21 mediates IL-6 or TNF-α production and NLRP3, ASC, and caspase-1 expression by targeting A20. Therefore, miR-21 specifically promotes NLRP3 inflammasome activation and the NF-κB signaling pathway by inhibiting A20. The results also suggest that miR-21 participates in a positive feedback loop to control NF-κB signaling in macrophages.

It has been reported that A20 inhibits NLRP3 inflammasome activation^31^. This study showed that miR-21 deficiency not only prevents IL-1β secretion and caspase-1 activation but also prevents caspase-1 cleavage of GSDMD to induce pyroptosis by targeting A20. Moreover, miR-21 deficiency suppresses NLRP3-mediated, but not AIM2-mediated caspase-1 activation. These results suggest that miR-21 knockout can negatively regulate caspase-1 activation through NLRP3 inflammasomes rather than AIM2 inflammasomes. NLRP3 inflammasome activation is strictly controlled to varying degrees in wild-type macrophages. A priming signal (also called the first signal and usually provided by TLR) upregulates *Nlrp3* and pro-IL-1b expression levels through the transcription factor NF-κB^22^, and A20 negatively regulates LPS-induced NF-κB activation^4–7^. We found that miR-21 deficiency negatively regulates LPS-induced NF-κB activation by promoting A20, leading to reduced secretion of cytokines (IL-6 and TNF) dependent on NF-κB in miR-21 KO macrophages. Thus, these data demonstrate that the inhibitory effect of miR-21 deficiency on NLRP3 inflammasome expression is dependent on NF-κB through promotion of A20. After the priming phase, the second signal is the activation signal, which initiates assembly of several protein complexes, including NLRP3, ASC, and pro-caspase-1, by regulating the formation of the ASC pyroptosome and splicing of caspase-1 into its active form. In this study, we demonstrated that miR-21 deficiency inhibited ASC oligomerization. Furthermore, we found that A20 knockdown promoted ASC oligomerization in WT cells, but this effect was reversed in miR-21 KO cells. These data demonstrate that miR-21 regulates ASC pyroptosome formation to activate the NLRP3 inflammasome through targeting of A20.

GSDMD has been identified as a universal substrate for caspase-1 and caspases-4/5/11. Ligands of various canonical inflammasomes can activate caspase-1, while caspases-4/5/11 directly recognizes bacterial LPS, and both activities can trigger pyroptosis^32^. Cleavage of GSDMD by inflammatory caspases critically determines pyroptosis by releasing the cleaved GSDMD-N domain, leading to induction of pyroptosis activity^32^. Despite its crucial role in septic shock, the mechanism of miR-21-induced pyroptosis is unclear. In this study, we found that A20 inhibits the ASC pyroptosome, which induced NLRP3 inflammasome assembly and activation of caspase-1-mediated GSDMD cleavage, thus preventing pyroptosis. We further found that miR-21 deficiency reversed the above results through targeting of A20, which prevents NLRP3 inflammasome activation and pyroptosis.

In this study, we found that miR-21 inhibits NLRP3 inflammasome activation in mouse macrophages and human THP-1 cells and primary PBMCs. The decreased activation of caspase-1 prevents cleavage of GSDMD, thus inhibiting pyroptosis in human THP-1 cells and primary PBMCs with miR-21 knockdown. Hence, miR-21 may act as a novel therapeutic target and biomarker in human septic shock.

In summary, our study provides a mechanistic explanation of miR-21-induced modulation of NLRP3 inflammasome-mediated immune responses and GSDMD-mediated pyroptosis in septic shock. Intriguingly, our results suggest that miR-21 may be an intrinsic positive regulator of abnormal NLRP3 inflammasome-induced septic shock. We observed that human NLRP3 inflammasomes are inhibited by miR-21 knockdown, suggesting miR-21 as a potential therapeutic target for treatment of inflammatory diseases and septic shock.

## Materials and methods

### Animals

C57BL/6 mice (6–8 weeks old) were obtained from the Academy of Military Medical Science (Beijing, China). Fei Gao’s lab (Institute of Zoology, Chinese Academy of Sciences, Beijing) kindly provided miR-21^−/−^ mice, which were generated using CRISPR-Cas9 technology. These animals were maintained in a specific pathogen-free animal facility at the Experimental Animal Center of Tianjin Medical University (Tianjin, China). The experiments were approved by the Animal Ethics Committee of Tianjin Medical University (Tianjin, China) and were carried out in accordance with animal care guidelines.

### Plasmids and reagents

Lipopolysaccharide (LPS; *E. coli* O111:B4, L3024), ATP and nigericin were purchased from Sigma-Aldrich (St. Louis, USA). Cytokines were measured using murine IL-1β, IL-18, IL-6, and TNF-α ELISA kits purchased from MultiSciences Biotech (Lianke Bio, Hangzhou, China). Cell death was determined with an LDH assay performed using a CytoTox 96 Non-Radioactive Cytotoxicity Assay kit (Promega, USA). The FUIGW-Flag-mgsdmd plasmid was kindly provided by Shao Feng (National Institute of Biological Sciences, Beijing, China).

### Cell culture and transfection

The bone marrow-derived macrophage preparation methods were as follows: bone marrow cells were extracted from femurs and tibias of wild-type and miR-21^−/−^ mice. Cells were then cultured at 2 × 10^6^ cells/ml in 10-cm cell culture plates in the DMEM supplemented with 10 ng/ml murine M-CSF. Cells were cultured for 6 days, and fresh medium was added every 3 days. On day 6, cells were collected for experiments. Macrophages were primed with 500 ng/ml LPS for 4 h before stimulation with 5 mM ATP and 20 μM nigericin for 30 min. The THP-1 human macrophage line was purchased from the Cell Resource Center of Peking Union Medical College. Cells were cultured in the RPMI-1640 medium supplemented with 10% FBS (HyClone, GE, USA) and antibiotics (100 IU/ml penicillin and 100 µg/ml streptomycin). Human peripheral blood mononuclear cells were prepared as we previously reported^33^. Briefly, 3 × 10^6^ cells were resuspended in 1 ml of RPMI-1640 medium containing 100 IU/ml penicillin, 100 μg/ml streptomycin, 2 mM L-glutamine, 1 mM pyruvate, and 10 ng/ml human M-CSF. Cells were cultured for 6 days, and fresh medium was added every 3 days. On day 6, the cells were collected for experiments. To achieve stable Flag expression, lentiviral plasmids containing the required genes, together with the packaging plasmids pspax2 and pmd2g, were used to transfect 293T cells at a ratio of 5:3:2. After transfection for 48 h and 72 h, the supernatants were collected and used to infect BMDMs for an additional 48 h. GFP-positive cells were sorted via flow cytometry for subsequent experiments.

### Quantitative real-time PCR

According to the manufacturer’s instructions, RNA was extracted using Trizol reagent (Invitrogen, Carlsbad, USA). After RNA purification, contaminated genomic DNA was removed using DNA enzymes. Random hexamers and M-MLV reverse transcriptase (Promega, Madison, USA) were used to reverse transcribe RNAs into cDNA. Takara (Takara, Japan) provided all other reverse transcription reagents. Genewiz (Suzhou, China) synthesized the gene-specific primers. In accordance with the manufacturer’s instructions, SYBR Green mix (Takara, Japan) was used for relative quantitative real-time PCR. Each reaction was performed on an ABI PRISM 7500 Fast Real-Time PCR System (Applied Biosystems Inc., Foster City, California, USA), and repeated in triplicate. The following primer pairs were used: for mouse *Nlrp3*, 5′-ATTACCCGCCCGAGAAAGG-3′ and 5′-TCGCAGCAAAGATCCACACAG-3′ mouse *Asc*, 5′-CTTGTCAGGGGATGAACTCAAAA-3′ and 5′-GCCATACGACTCCAGATAGTAGC-3′ mouse *Casp1*, 5′-ACAAGGCACGGGACCTATG-3′ and 5′-TCCCAGTCAGTCCTGGAAATG-3′ and mouse *Tnfaip3*, 5′-GAACAGCGATCAGGCCAGG-3′ and 5′-GGACAGTTGGGTGTCTCACATT-3′.

### 3′-UTR luciferase assays

The WT or mutant *Tnfaip3* 3′-UTR was amplified via PCR and cloned into a pmirGLO Dual-luciferase miRNA Target Expression Vector (Promega, USA). Lipo3000 transfection reagent was used to cotransfect 293T cells with miR-21 mimic and pmirGLO Dual-luciferase 3′-UTR vector. At 48 h post transfection, the cells were harvested and assayed using a dual-luciferase reporter assay system (Promega, USA).

### Small interfering RNAs and transfection

miR-21 mimic, inhibitor, and control siRNA were synthesized by RiboBio (Guangzhou, China). The siRNA for *Tnfaip3* knockdown had the following sequence: 5′-GCUGUGAAGAUACGAGAGAUU-3′ (sense sequence). According to the manufacturer’s instructions, cells were treated with siRNAs (final concentration of 25 nM) and harvested 48 h after siRNA treatment using Lipofectamine RNAiMAX (Invitrogen, Carlsbad, CA, USA).

### Western blot analysis

Lysis buffer containing 10 mM Tris-buffer (pH 7.6), 1% Triton X-100, 1% phosphatase inhibitor cocktail, and 1 mM PMSF was used to lyse cells. Cell lysates were then boiled in SDS sample buffer and resolved on a 10% SDS-PAGE gel. Immunoblots were incubated overnight with primary antibodies against NLRP3 (AG-20B-0014-C100, Adipogen, CH), ASC (AG-25B-0006, Adipogen, CH), mouse caspase-1 (AG-20B-0042-C100, Adipogen, CH), human caspase-1 (AG-20B-0048-C100, Adipogen, CH), IkBa (4814, Cell Signaling, USA), phospho-IkBa (2859, Ser32) (Cell Signaling, USA), A20 (23456-1-AP, Proteintech, USA), IL-1β (12242, Cell Signaling, USA) human GSDMD (sc-81868, Santa Cruz Biotechnology, USA), mouse GSDMD (ab209845, Abcam, USA), and GAPDH (10494-1-AP, Proteintech, USA). Immunoblots were examined using an ECL detection reagent (Millipore Corporation, Billerica, MA, USA).

### Flow cytometry

Cells were washed from the peritoneal cavities of mice injected intraperitoneally with LPS and resuspended in PBS containing 1% FBS for FACS analysis. According to the manufacturer’s instructions, anti-CD11b, anti-F4/80, anti-MHC II, and anti-CD206 antibodies were used for fluorescent staining. Isotype antibody controls were used to exclude nonspecific staining. All flow-cytometric antibodies were obtained from Ebioscience. The data were obtained with a FACSCanto II flow cytometer (BD Biosciences, USA) and analyzed using FlowJo software (Tree star, Ashland, OR).

### Microscopy imaging of cell death

To examine cell death morphology, cells were treated in a 12-well plate according to the instructions for static imaging of live cells. An Olympus IX71 microscope was used to capture static bright-field images of pyroptotic cells. For ASC pyroptosome formation, macrophages were seeded overnight on 35-mm glass-bottom culture dishes. The cells were stimulated and stained with an anti-ASC antibody and DAPI, as described above. After stimulation, the cells were fixed with 4% paraformaldehyde for 20 min and then permeabilized with Triton X-100. The cells were then incubated with an anti-ASC antibody for 1 h and donkey anti-rabbit antibody conjugated to Alexa Fluor 488 as the secondary antibody (Proteintech, USA). Finally, the cells were stained with DAPI. An Olympus FluoView FV1000 microscope equipped with a ×60 objective was used for cell imaging. The images were processed using ImageJ. All image data shown are representative of at least three randomly selected fields.

### ASC pyroptosome detection

As previously reported, we detected the formation of ASC pyroptosomes^29^. BMDMs were cultured in six-well plates at a concentration of 2 × 10^6^ cells/ml and treated with different stimulants. Cells were centrifuged and resuspended in 0.5 ml of ice-cold buffer containing 20 mM HEPES-KOH (pH 7.5), 150 mM KCl, 1% NP-40, 0.1 mM PMSF, and protease inhibitors, and were lysed in a microcentrifuge tube via shearing 20 times with a 21-gauge needle. The cell lysate was centrifuged at 5000 × *g* for 10 min at 4 °C. The supernatant was collected and diluted with 1 volume of buffer and centrifuged for 8 min at 5000 × *g* to granulate ASC oligomers. Next, the resuspended pellets were cross-linked with fresh DSS (4 mM, dissolved in DMSO) for 30 min and centrifuged for 10 min at 5000 × *g*. The cross-linked pellets were resuspended in 30 μl of SDS sample buffer and separated on a 10% SDS-PAGE gel. An anti-mouse ASC antibody was used for western blotting.

### Histopathology

Spleens, livers, and kidneys from wild-type and miR-21^−/−^ mice were perfused with 4% paraformaldehyde infused through the heart and then dissected and fixed overnight. The spinal cord was embedded in paraffin and sectioned (5–10 µm) for hematoxylin and eosin staining (H&E). The degree of inflammatory cell infiltration was analyzed via routine histology and quantified using ImageJ software.

### In vivo septic shock model

To induce cytokine secretion in vivo, 8-week-old female mice were intraperitoneally injected with LPS. At 6 h after the injection, 0.8 ml of PBS containing 1% FBS was injected into the peritoneal cavity, and then, the fluid was separated. An ELISA was used to measure the IL-1β level in peritoneal lavage fluid and IL-18 level in serum. To induce septic shock, LPS was injected into the peritoneal cavity of mice, and then the mouse health status was monitored at specific intervals.

### Statistics

GraphPad Prism 6.0 software was used for data analysis. The data are presented as the mean ± SD of triplicate measurements in a representative experiment. Statistical analysis was performed using an unpaired two-tailed *t* test. *P* < 0.05 was considered statistically significant.
